# Antibiotic Tolerance and Treatment Outcomes in Cystic Fibrosis Methicillin-Resistant Staphylococcus aureus Infections

**DOI:** 10.1128/spectrum.04061-22

**Published:** 2022-12-15

**Authors:** Kuan-Yi Lu, Nikki J. Wagner, Amanda Z. Velez, Agathe Ceppe, Brian P. Conlon, Marianne S. Muhlebach

**Affiliations:** a Department of Microbiology and Immunology, University of North Carolina at Chapel Hill, Chapel Hill, North Carolina, USA; b Marsico Lung Institute, University of North Carolina at Chapel Hill, Chapel Hill, North Carolina, USA; c Department of Pediatrics, University of North Carolina at Chapel Hill, Chapel Hill, North Carolina, USA; University of Pittsburgh

**Keywords:** antibiotic tolerance, MRSA, cystic fibrosis, persistence, treatment outcome, *Staphylococcus aureus*

## Abstract

Methicillin-resistant Staphylococcus aureus (MRSA) is highly prevalent in U.S. cystic fibrosis (CF) patients and is associated with worse clinical outcomes in CF. These infections often become chronic despite repeated antibiotic therapy. Here, we assessed whether bacterial phenotypes, including antibiotic tolerance, can predict the clinical outcomes of MRSA infections. MRSA isolates (*n* = 90) collected at the incident (i.e., acute) and early infection states from 57 patients were characterized for growth rates, biofilm formation, hemolysis, pigmentation, and vancomycin tolerance. The resistance profiles were consistent with those in prior studies. Isolates from the early stage of infection were found to produce biofilms, and 70% of the isolates exhibited delta-hemolysis, an indicator of *agr* activity. Strong vancomycin tolerance was present in 24% of the isolates but was not associated with intermediate vancomycin susceptibility. There were no associations between these phenotypic measures, antibiotic tolerance, and MRSA clearance. Our research suggests that additional factors may be relevant for predicting the clearance of MRSA.

**IMPORTANCE** Chronic MRSA infections remain challenging to treat in patients with cystic fibrosis (CF). The ability of the bacterial population to survive high concentrations of bactericidal antibiotics, including vancomycin, despite lacking resistance is considered one of the main reasons for treatment failures. The connection between antibiotic tolerance and treatment outcomes remains unexplored and can be crucial for prognosis and regimen design toward eradication. In this study, we measured the capacity of 90 MRSA isolates from CF patients to form vancomycin-tolerant persister cells and evaluated their correlation with the clinical outcomes. Additionally, various traits that could reflect the metabolism and/or virulence of those MRSA isolates were systematically phenotyped and included for their predictive power. Our research highlights that despite the importance of antibiotic tolerance, additional factors need to be considered for predicting the clearance of MRSA.

## INTRODUCTION

The incidence rate for methicillin-resistant Staphylococcus aureus (MRSA) lung infection is about 8% at cystic fibrosis (CF) centers in the United States (1). The majority of these patients develop persistent infections, with pancreatic insufficiency, CF-related diabetes, and Pseudomonas aeruginosa coinfection being relevant risk factors (2). As chronic MRSA infection is associated with worse patient survival rates compared to all other CF infections and failure to recover from exacerbations, understanding of the factors driving persistent MRSA infections in CF is much needed (3, 4).

As a standard procedure to determine antibiotic treatment regimens, antimicrobial susceptibility testing (AST) has been shown useful in predicting clinical outcomes in acute infections (5). However, the standard AST has poor predictive power in chronic and recurrent infections and is not associated with better treatment success in CF (6). Other tests, such as biofilm AST, have also failed to improve predictive power in CF (7). It is thus likely that other factors are responsible for dictating treatment outcomes. Unlike resistance, antibiotic tolerance measures the ability of a bacterial population to survive exposure to bactericidal antibiotics. In this scenario, the MICs of the antibiotics remain the same, and thus tolerance cannot be detected by ASTs. Importantly, highly tolerant subpopulations of bacteria, also known as persister cells, have been implicated in antibiotic treatment failure and can promote the evolution of antibiotic resistance (8–11). Evidence suggests that P. aeruginosa has evolved an increased capacity to produce persister cells in patients with CF (12). Whether antibiotic tolerance and persister formation link to treatment failures in CF MRSA infections has yet to be investigated.

In this study, we examined whether antibiotic tolerance is associated with clearance of MRSA following initial MRSA-positive respiratory cultures. Additional phenotypic traits, including resistance, growth rates, biofilm formation, hemolysis, pigmentation and postantibiotic lag times were characterized to identify novel predictors of treatment outcomes.

## RESULTS

### Study population.

Enrolled subjects (*n* = 57) with matched clinical data and MRSA isolates (a total of 90 isolates) were included in this study. The subject age was 13.7 ± 8.9 years (mean ± SD) (16 adults), with an estimated lung function median forced expiratory volume in 1 s (FEV_1_) value of 81.2 ± 23.2% predicted per Global Lung Function Initiative (GLI) reference (*n* = 46). Of the 57 subjects, 10 had coinfection with Pseudomonas aeruginosa, and 16 were on CF transmembrane conductance regulator (CFTR) modulator therapy. Ten of the 57 subjects had only one culture documented. Among the 47 subjects with repeat documented cultures, 15 (32%) had cleared infection, and 32 (68%) remained MRSA positive. There was no significant difference in persistence between the groups with and without P. aeruginosa coinfection—69% of the P. aeruginosa-negative patients and 63% of the P. aeruginosa-positive patients developed MRSA persistence (*P* = 0.69). Among the 90 isolates (incident and repeat), 34 had prior exposure to antibiotics within 14 days before collection, with 29 isolates with at least one oral antibiotic course and 15 exposed to inhaled antibiotics (e.g., tobramycin) (see Data set S1 in the supplemental material). Oral antibiotics included azithromycin (*n* = 15), amoxicillin-clavulanate (*n* = 5), trimethoprim-sulfamethoxazole (*n* = 4), tetracycline (*n* = 3), and/or combinations thereof (*n* = 5) (Data set S1). Additionally, four patients having pulmonary exacerbations also received intravenous (i.v.) vancomycin therapy at some point. Ten patients with P. aeruginosa coinfection received a combination of both oral and inhaled antibiotics. Notably, antibiotic exposure did not differ between the incident and repeat MRSA isolates (*P* = 0.46).

### Phenotypic profiling of MRSA isolates from cystic fibrosis patients.

**FIG 1:**
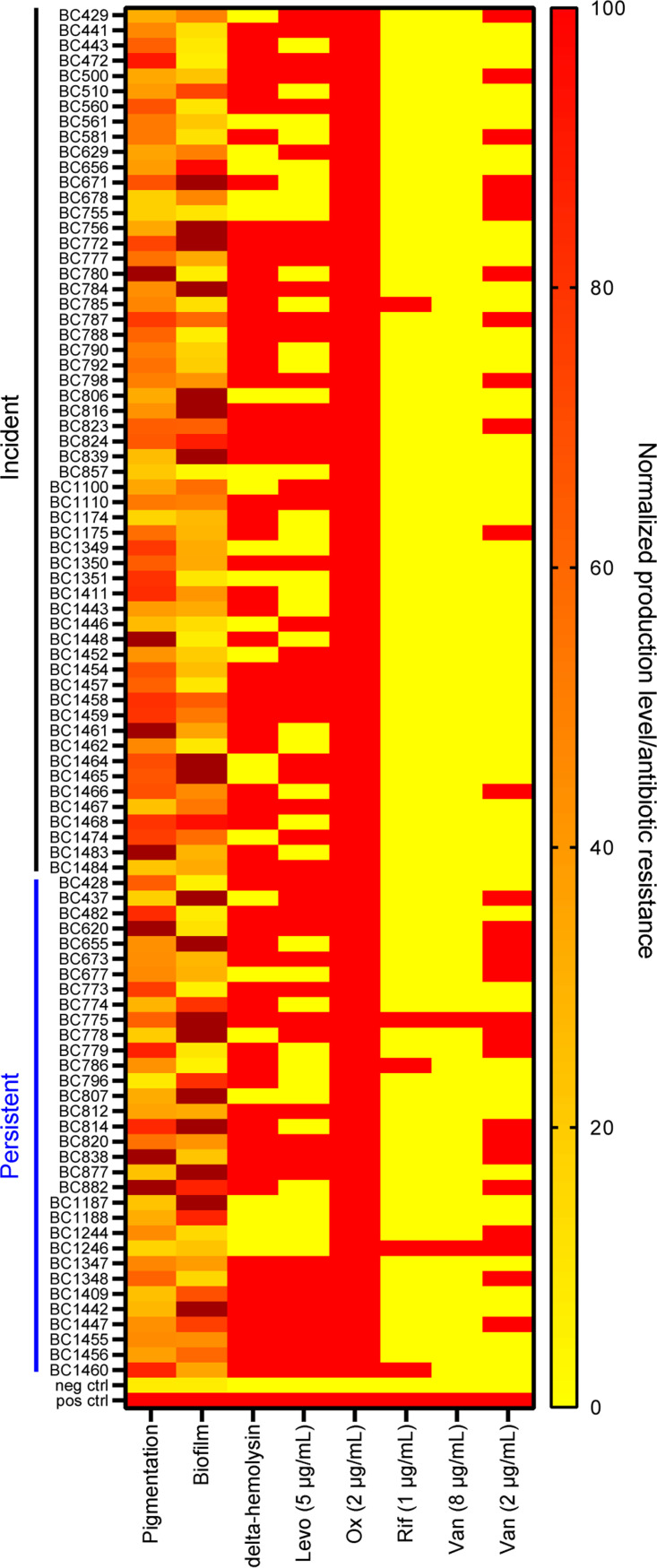
Phenotypic profiling of 90 MRSA isolates from cystic fibrosis patients. Carotenoid pigment and biofilm production of the clinical isolates were measured and normalized to those of the positive controls (HG003 and a biofilm-producing strain, BC1381). Those producing more pigment and biofilm than the positive controls are shown in brown. Strains RN4220 and USA300 LAC were included as the negative controls for pigmentation and biofilm production, respectively. Clinical isolates with delta-hemolysin activity and/or resistance to 5 μg/mL levofloxacin (Levo), 2 μg/mL oxacillin (Ox), 1 μg/mL rifampicin (Rif), and 2 μg/mL and 8 μg/mL vancomycin (Van) were identified (red). Wild-type HG003 and its *agrC* mutant (*agrC*::erm) were the positive and negative controls for delta-hemolysin activity.

All the isolates were confirmed to be oxacillin resistant at an MIC of ≥2 μg/mL, and 94% and 78% were resistant to oxacillin at 4 and 10 μg/mL, respectively. Consistent with previous reports (13, 14), the resistance rate to levofloxacin was high (57% with an MIC value of ≥5 μg/mL) (Fig. 1; Data set S1). As expected, 98% of the isolates were vancomycin sensitive (MIC, ≤8 μg/mL), although 27 (30%) of them had reduced susceptibility (MIC, ≥2 μg/mL) (15). We also tested rifampicin, an antibiotic with potent bactericidal activity against S. aureus. Antibiotic resistance was less frequent among the incident than repeat isolates for rifampicin (20% versus 80%, *P* = 0.03) and vancomycin (44% versus 56%, *P* = 0.01) but not for levofloxacin (62% versus 38%, *P* = 0.6). Three independent isolates (BC775, BC1246, and BC1460) exhibited resistance to at least two antibiotics in addition to oxacillin (Fig. 1; Data set S1).

Next, we profiled the phenotypic traits that reflect the metabolism and/or virulence of these MRSA isolates. These traits include biofilm formation and carotenoid pigment production, where biofilm facilitates MRSA colonization in CF lungs, and pigmentation has been linked to virulence (16–18). Colorimetric analysis revealed that 20% of the isolates generated more biofilm matrix compared to a previously identified biofilm-producing strain (Fig. 1; Fig. S1). In comparison with a reference strain, HG003, which produces a high level of carotenoid pigment, only 7% of the isolates showed higher pigmentation, while half of the isolates (45 of 90) had <50% pigment levels of strain HG003 (Fig. 1; Fig. S2). Additionally, we investigated the expression of delta-hemolysin (delta-toxin) as a proxy for Agr activity, which has been linked to various virulence traits (19). We found that 73% of the isolates were delta-hemolysin positive, hinting at their capacity to express virulence factors (Fig. 1, Fig. S3, and Data set S1).

**FIG 2:**
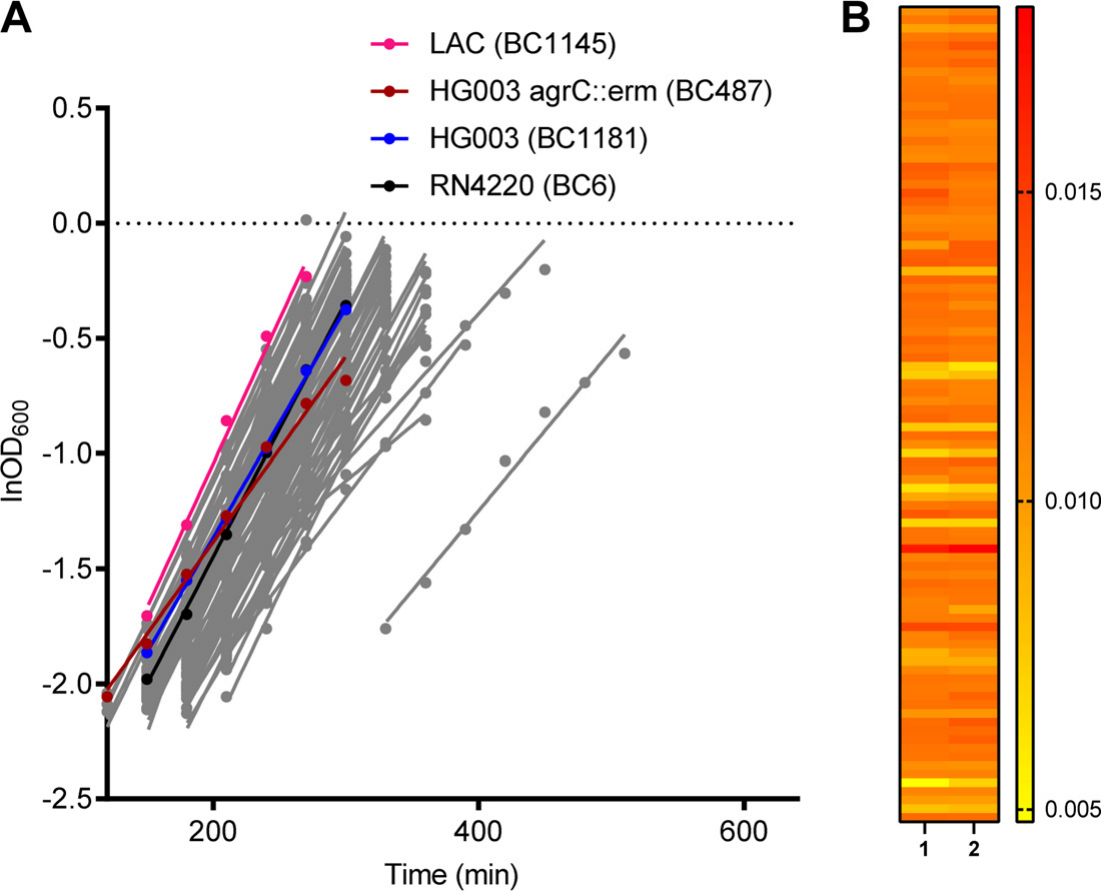
MRSA isolates from cystic fibrosis patients display various growth rates. Planktonic growth of the clinical isolates and reference laboratory strains was measured. (A) Exponential growth of the 90 isolates. Representative data of two biological replicates are shown. (B) Heat map showing the consistent growth rates (ΔlnOD_600_/min) between two independent assays.

Another trait that has been associated with treatment efficacy is the bacterial growth rate. The growth rates ranged from 0.006 to 0.019 ΔlnOD_600_/min with a median value of 0.012 ΔlnOD_600_/min (the increase in the natural logarithm of the OD_600_ per min in the linear exponential growth phase). Compared to the well-characterized MRSA strain LAC (growth rate, 0.0125 ΔlnOD_600_/min), four isolates (BC812, BC1175, BC1246, and BC1484) can be classified as slow-growing bacteria, with growth rates lower than 0.0073 ΔlnOD_600_/min (the growth rate of LAC minus 3-fold standard deviation) (Fig. 2). One isolate (BC1349) was classified as a fast-growing bacterium, with a growth rate higher than 0.018 ΔlnOD_600_/min (the growth rate of LAC plus a 3-fold standard deviation).

### Antibiotic tolerance.

**FIG 3:**
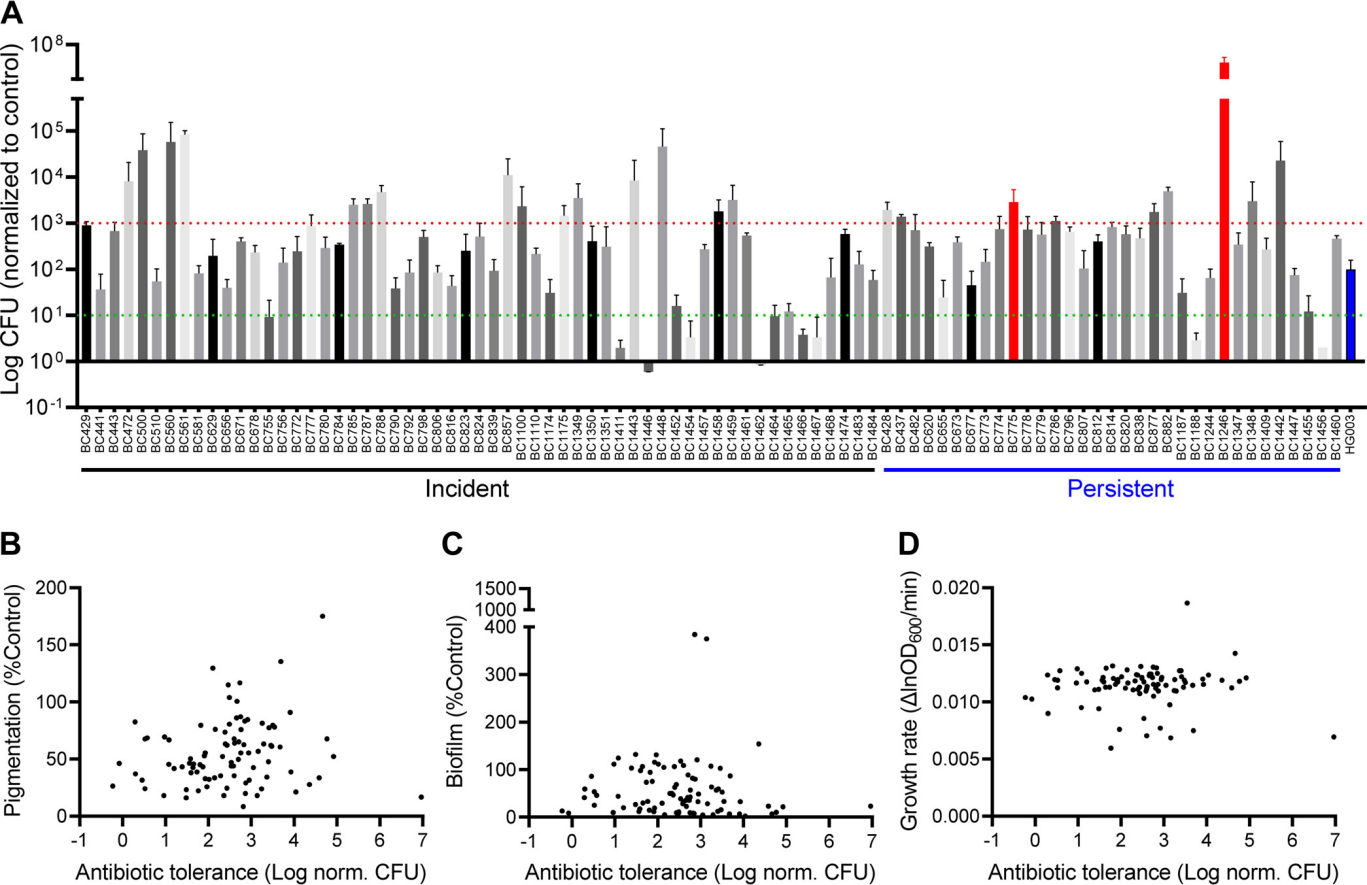
Antibiotic tolerance to vancomycin is not correlated with the growth rate of MRSA isolates or their capacity to produce pigment and biofilm. (A) Antibiotic tolerance of the clinical MRSA isolates. The isolates were treated with 20 μg/mL vancomycin for 16 h, and the surviving cells were enumerated. The CFU were normalized to those of strain HG003 (blue bar). Vancomycin-resistant isolates (MIC, ≥8 μg/mL) are indicated in red. Isolates showing 10-fold more tolerant cells (above the red dotted line) and 10-fold less tolerant cells (beneath the green dotted line) were considered highly tolerant and highly susceptible isolates, respectively. The bars represent the mean ± SD (*n* = 3). Antibiotic tolerance of the clinical isolates was not correlated with pigmentation (B), biofilm production (C), or their growth rates (D) [Pearson correlation coefficient, *r* = −0.1382 (B), −0.05998 (C), and −0.2749 (D)].

To probe the possible role of tolerance in predicting treatment outcomes, we first quantified antibiotic tolerance and evaluated its relationship with the aforementioned phenotypic traits. Although not all patients received the same treatment regimen, current evidence show that persisters are phenotypically tolerant to multiple different antibiotics (20–23). Here, we used vancomycin to probe the persister-forming capacity of the MRSA isolates, given its clinical relevance (as the first-line treatment for patients requiring i.v. therapy) and the general absence of resistance in our collection (Fig. 1; MIC, <8 μg/mL). We exposed each MRSA isolate to 20 μg/mL vancomycin, followed by diluting and plating the cultures on antibiotic-free tryptic soy agar to enumerate the number of bacteria that survived vancomycin treatment. Of the 88 isolates (not including the two vancomycin-resistant isolates), 24% were 10-fold more tolerant than the reference strain HG003, while 11% were 10-fold less tolerant than the reference strain (Fig. 3A; Fig. S4). The frequency of low tolerance versus high tolerance did not differ between the incident and repeat isolates (*P* = 0.49), nor between groups with different vancomycin susceptibility (MIC < 2 μg/mL versus MIC ≥ 2 μg/mL) (*P* = 0.65). We did not observe correlations between vancomycin tolerance and pigmentation, biofilm production, or the growth rate (Fig. 3B to D). As such, additional factors, or more likely a combination of multiple factors, might be needed to predict antibiotic tolerance.

Another parameter to assess antibiotic tolerance is the postantibiotic effect (PAE)—the time it takes to resume growth after removing antibiotic pressure. As shown in Fig. S5, most isolates had similar lag times when they were subcultured from the stationary phase without antibiotic treatment, while intriguingly, they exhibited significantly different PAEs. These data revealed a wide range of capacities of the MRSA isolates to recover from antibiotic stress, where some isolates resumed growth within 10 h of antibiotic exposure, and some required >36 h to recover. Further analysis of diversity from single patients, before and after antibiotic therapy, is required to determine the significance of PAE variability and antibiotic treatment failure.

### Lack of association between bacterial phenotypes and clinical outcomes.

We assessed whether the MRSA phenotypes were associated with persistent infection in the 47 subjects with documented follow-up visits. There were no differences in antibiotic susceptibility, hemolysis, pigmentation, growth rates, or biofilm formation in either single or multivariate analyses (Table S1). No significant difference in vancomycin tolerance was detected between patients who cleared versus those having continued MRSA-positive cultures (*P* = 0.2). Additionally, we explored the impact of sample types (i.e., sputum versus oropharyngeal swab samples) and adjusted the data for subject age and FEV_1_, given that older subjects and those with lower FEV_1_ were more likely to expectorate. Consistently, no significant differences were detected for clearance of MRSA or any of the MRSA phenotypes (Table S1).

## DISCUSSION

Increasing evidence suggests that antibiotic tolerance and persister cell formation contribute to treatment failure in bacterial infections (8–10). To test the hypothesis that antibiotic tolerance dictates the treatment outcomes in CF MRSA infections, we measured vancomycin tolerance and various phenotypes of 90 MRSA isolates from 57 CF patients. Vancomycin tolerance was variable between those isolates, yet a quarter of the isolates were highly tolerant compared to the reference strain. We found that vancomycin tolerance was not associated with the clearance of MRSA. Other phenotypic traits, alone or in combination with antibiotic tolerance, also did not show predictive power on treatment success. Our data suggest that additional factors such as the bacterial metabolic state (the amount of ATP present) and the heterogeneity of the bacterial population in patients may need to be taken into account (24–26). Interestingly, 40% of the isolates exhibited variable PAEs that differed by >10 h (e.g., a postantibiotic lag time of 7.7 h and >36 h for BC1411, determined in two independent experiments) (see Fig. S5A in the supplemental material). This variability was likely due to heterogeneity in the population, which emerged once exposed to vancomycin (27). As bacteria in the lag phase are tolerant to growth-dependent antibiotics such as vancomycin, these findings could indicate that lung adaptation and/or frequent antibiotic exposure results in a population with a highly heterogenous PAE, increasing the likelihood of survivors during intermittent antibiotic dosing. Further studies to probe PAE heterogeneity are required to determine the relevance of antibiotic treatment failure in patients.

It is also possible that antibiotic tolerance is contributing to treatment outcomes, but measuring the trait under *in vitro* conditions does not translate to tolerance during infection. We have recently shown that during host cell interaction, antibiotic tolerance can be strongly and equally induced in a variety of clinical isolates with differing *in vitro* antibiotic tolerance profiles (28). Additionally, S. aureus can reside in mammalian cells, including macrophages, and the intracellular niche has been shown to shape persister formation (8, 29). These findings suggest that tolerance may be dictating outcomes in patients, but measuring tolerance under homogenous, artificial conditions is unlikely to identify clinically relevant phenotypes. Instead, it is likely necessary to measure antibiotic tolerance *in vivo*, or under conditions that better mimic the infection microenvironment. On top of this, the host factors may also be critical in determining clinical outcomes. For example, despite being difficult to measure, mucus obstruction and the degree of preexisting airway inflammation in the patients could be relevant determinants for bacterial clearance.

In conclusion, MRSA isolated from CF patients showed diverse phenotypes, ranging from biofilm formation to their ability to tolerate antibiotics. Despite not being vancomycin resistant, many isolates, to various degrees, can tolerate a high concentration of vancomycin even early in the course of the infection. This tolerance was not correlated with bacterial growth rate nor their abilities to produce biofilm, pigment, or delta-hemolysin. Single and multifactorial analyses did not reveal a significant correlation between antibiotic tolerance and treatment outcomes. Given the high variability in the production of virulence factors and antibiotic tolerance, additional factors, including bacterial population heterogeneity, postantibiotic effect, and, in particular, antibiotic tolerance in the infection microenvironment, might need to be considered to effectively predict outcomes in patients.

## MATERIALS AND METHODS

### Subjects and isolate collection.

Bacterial isolates were collected from CF patients with incident MRSA detection. These isolates were collected across four study sites (University of North Carolina, Oregon Health and Science University, John Hopkins University, and Boston Children’s Hospital). Respiratory cultures, including deep pharyngeal swabs, expectorated sputum, or bronchoalveolar lavage, were cultured according to the CF microbiology guidelines (30). Three random colonies were selected, subcultured, and kept at −80°C for the following experiments. Clinical information was collected and uploaded to the REDCap database. This study was approved by the ethical committee of each institute and was exempt from patient consent, given the less than minimal risk.

### MRSA isolates and growth assay.

Stationary-phase cultures of the S. aureus isolates were diluted in tryptic soy broth (TSB) at 1:1,000 in a 96-well clear plate with a low evaporation lid (Corning). Bacterial growth was monitored by measuring the OD_600_ values every 30 min for 36 h at 37°C with continuous shaking using a Synergy H1 microplate reader (BioTek). The exponential growth was determined by fitting data to a standard growth curve equation using Prism software (GraphPad).

### Pigmentation measurement.

S. aureus isolates were cultured in 1 mL TSB at 37°C for 24 h to reach the stationary phase. Bacteria were pelleted at 2,500 × *g* for 10 min, resuspended in 250 μL methanol, and incubated at 55°C for 3 min. After centrifugation at 2,500 × *g* for 5 min, 100 μL of the supernatants were transferred to a 96-well clear bottom plate (Corning). The absorbance of carotenoid pigments was determined by measuring their OD_465_ values using a Synergy H1 microplate reader. Strains HG003 and RN4220 were used as the positive and negative controls, respectively. Three independent cultures were examined to ensure reproducibility.

### Biofilm assay.

Overnight cultures of the S. aureus isolates were diluted in TSB at 1:200, followed by dispensing into a 96-well TC-treated plate (Corning) in eight replicates (100 μL/well). Plates were sealed with Breathe-EASIER membranes (Diversified Biotech) and incubated statically at 37°C for 24 h. Plates were washed with distilled water three times using a Nunc 12-channel Immuno Washer and dried at 65°C for 1 h. Each well was stained with 100 μL 0.4% (wt/vol) crystal violet at room temperature for 5 min and washed with distilled water three times to remove excess dye. Once air-dried, the biofilm-associated crystal violet was solubilized in 100 μL 5% (vol/vol) acetic acid, and their OD_492_ values were measured using a Synergy H1 microplate reader. The biofilm-producing S. aureus isolate BC1381 and strain USA300 LAC were the positive and negative controls, respectively. Three independent cultures were examined to ensure reproducibility.

### Delta-hemolysin assay.

Delta-hemolysin activities were assessed by cross-streaking overnight cultures of the S. aureus isolates perpendicularly to the reference strain RN4220, which only produces beta-hemolysin, on tryptic soy agar containing sheep blood (Thermo Scientific). Agar plates were incubated at 37°C for 18 to 24 h. Given that beta- and delta-hemolysins act synergistically, enhanced hemolysis can be observed at the intersection of a strain that secretes delta-hemolysin and the reference strain (19). The wild-type strain HG003 and its *agrC* mutant (*agrC*::erm) were used as the positive and negative controls, respectively. Three independent cultures were examined to ensure reproducibility.

### Antibiotic resistance and tolerance assays.

MRSA isolates were spotted onto tryptic soy agar containing 5 μg/mL levofloxacin (Alfa Aesar), 2 μg/mL oxacillin (ACROS Organics), 1 μg/mL rifampicin (Fisher Scientific), or 2 to 8 μg/mL vancomycin (Alfa Aesar), followed by incubation at 37°C for 24 h to monitor their resistance. The antibiotic concentrations used for determining the resistance were based on the CLSI criteria (31). To evaluate antibiotic tolerance among these isolates, vancomycin was chosen because of its clinical relevance and given that most isolates (88 out of 90) were sensitive to 8 μg/mL vancomycin. Briefly, stationary-phase cultures of the clinical isolates were diluted at 1:333 in TSB and treated with and without 20 μg/mL vancomycin at 37°C for 16 h. Each culture was serially diluted below the MIC of vancomycin and plated on tryptic soy agar to enumerate the number of persisters that survived vancomycin treatment. HG003 was included as a reference strain.

### Postantibiotic lag time measurement.

Stationary-phase cultures were diluted at 1:333 in TSB and treated with and without 20 μg/mL vancomycin at 37°C for 16 h. Next, each culture was diluted at 1:1,000 in TSB in a 96-well clear plate with a low evaporation lid to achieve a vancomycin concentration below 1 μg/mL (a subinhibitory concentration for all the isolates tested). Bacterial growth was monitored by measuring the OD_600_ values every 30 min for 36 h at 37°C with continuous shaking using a Synergy H1 microplate reader. The lag times were calculated using Gen5 software (BioTek).

### Statistical analysis.

Continuous data are reported as means ± SD unless otherwise stated. Tolerance data were log transformed. Groupwise comparisons were performed using Student’s *t* test and Fisher’s exact test for continuous and categorical variables, respectively. Multivariate analyses to assess the persistence of MRSA were conducted using repeated measure logistic regression to account for repeated visits per subject. Age and forced expiratory volume in the first second (FEV_1_%) as the percent predicted were then included as covariates in the repeated models. Analyses were conducted using JMP Pro V 15.1.0 and SAS 9.4.
