# Supplementary material for: Antibiotic Tolerance and Treatment Outcomes in Cystic Fibrosis Methicillin-Resistant Staphylococcus aureus Infections
Source: Microbiol Spectr. 2022 Dec 15;11(1):e04061-22. doi: 10.1128/spectrum.04061-22 (PMC9927320; doi:10.1128/spectrum.04061-22)
Supplement: Supplemental file 1 — Supplemental material. Download spectrum.04061-22-s0001.pdf, PDF file, 0.5 MB [file spectrum.04061-22-s0001.pdf]

Supplemental Material for

Antibiotic tolerance and treatment outcomes in cystic fibrosis MRSA infections

Kuan-Yi Lu, Nikki J. Wagner, Amanda Z. Velez, Agathe Ceppe, Brian P. Conlon, Marianne S. Muhlebach

Brian P. Conlon
Email: brian_conlon@med.unc.edu

Marianne S. Muhlebach
Email: marianne_muhlebach@med.unc.edu

This PDF file includes:

- Supplementary table 1
- Supplementary figure 1 to 5

Supplementary table 1. Correlation analysis of treatment outcomes and multiple phenotypes

Phenotype[1]	MRSA clearance	MRSA persistence	p-value[2]	p-value (repeated measures)	p-value (multivariate)[3]
Delta-hemolysin	17%	83%	0.32	0.61	0.99
Rifampicin resistance	20%	80%	1	0.69	0.90
Levofloxacin resistance	17%	83%	0.5	0.17	0.88
Vancomycin resistance	19%	81%	0.81	0.98	0.41
Growth rate	0.011 ± 0.0004	0.011 ± 0.0002	0.73	0.2536	0.1113
Biofilm formation	48.6 ± 16.2	60.1 ± 8.1	0.53	0.6180	0.7258
Lag time	1451 ± 128	1433 ± 64	0.90	0.7192	0.3350
Tolerance	2.8 ± 0.3	2.5 ± 0.14	0.39	0.6890	0.8537
Pigmentation	44.9 ± 6.6	54.8 ± 3.2	0.19	0.2775	0.1605

Footnotes: [1]Percentage of isolates expressing the phenotypes. [2]Repeated measure analysis. [3]Adjusted for age and FEV_1.

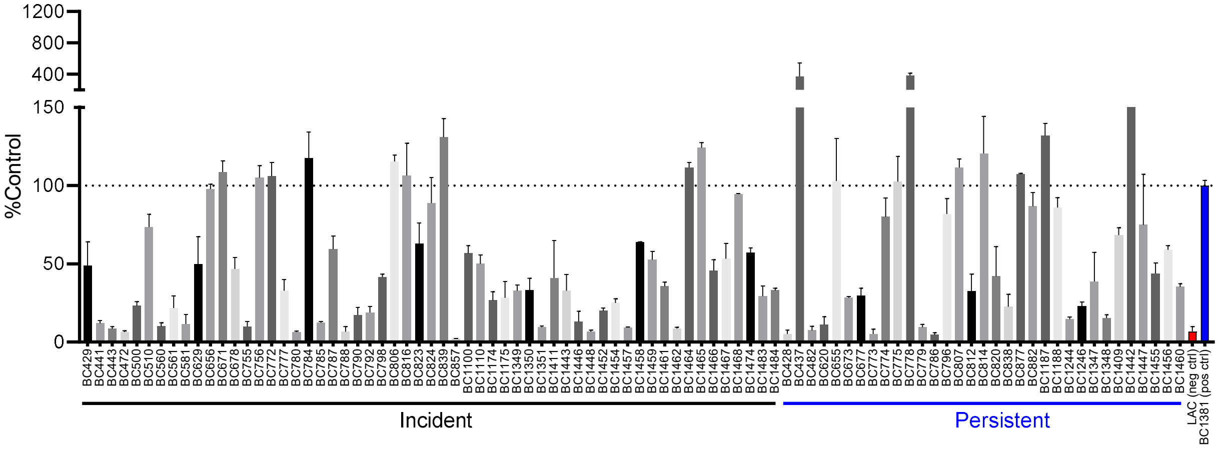

Supplementary figure 1. The biofilm-forming capacity varies among the clinical MRSA isolates. Biofilm formation by the clinical isolates was determined using a microtiter plate assay. The biofilm biomass values were normalized to that of a previously identified biofilm-producing isolate BC1381 (blue bar). Strain USA300 LAC (red bar) with limited biofilm formation was included for comparison. The bars represent mean ± SD (n = 3).

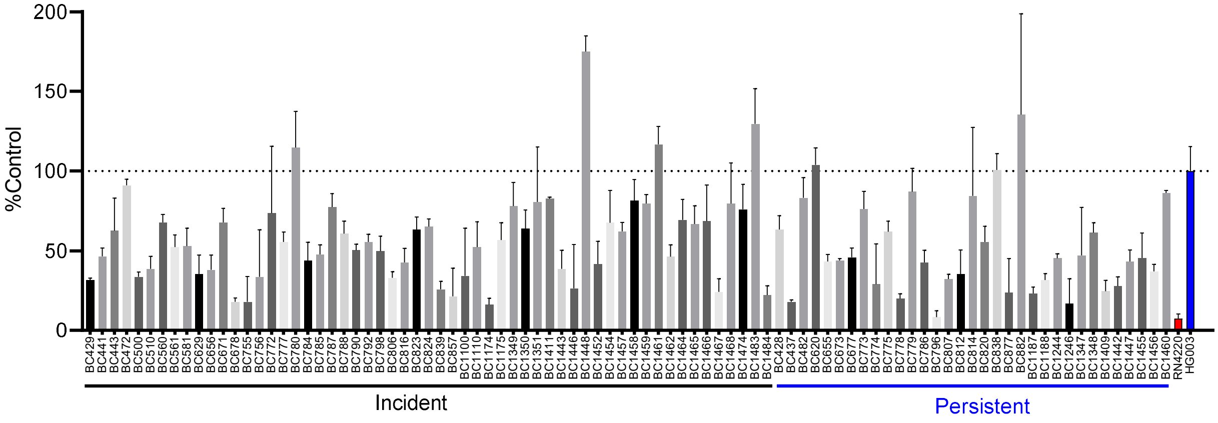

Supplementary figure 2. MRSA isolates from cystic fibrosis patients show diversity in pigment biosynthesis. The carotenoid levels were quantified and normalized to that of HG003 (blue bar). A non-pigmented strain RN4220 (red bar) was included for comparison. The bars represent mean ± SD (n = 3).

A B

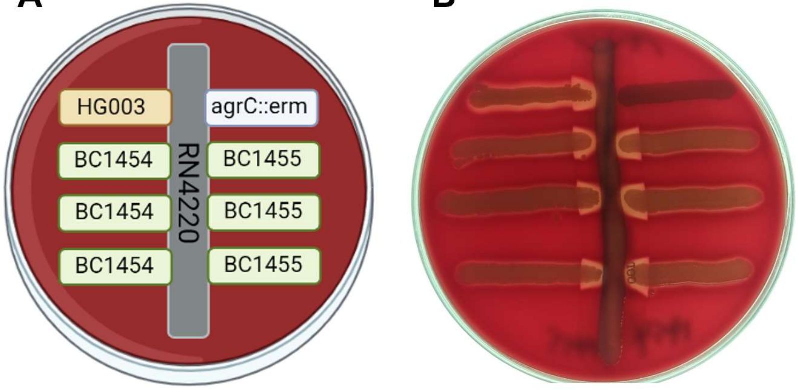

Supplementary figure 3. Many CF isolates produce delta-hemolysin. (**A**) Schematic of delta-hemolysin detection assay. Each isolate was streaked in triplicate against RN4220 which produces beta-hemolysin. Wild-type HG003 and its *agrC* mutant (*agrC*::erm) were the positive and negative controls for delta-hemolysin activity. (**B**) Representative data showing CF isolates (BC1454 and BC1455) generated delta-hemolysin. Production of delta-hemolysin can be visualized by enhanced hemolysis at the intersection of RN4220 and delta-hemolysin-producing strains.

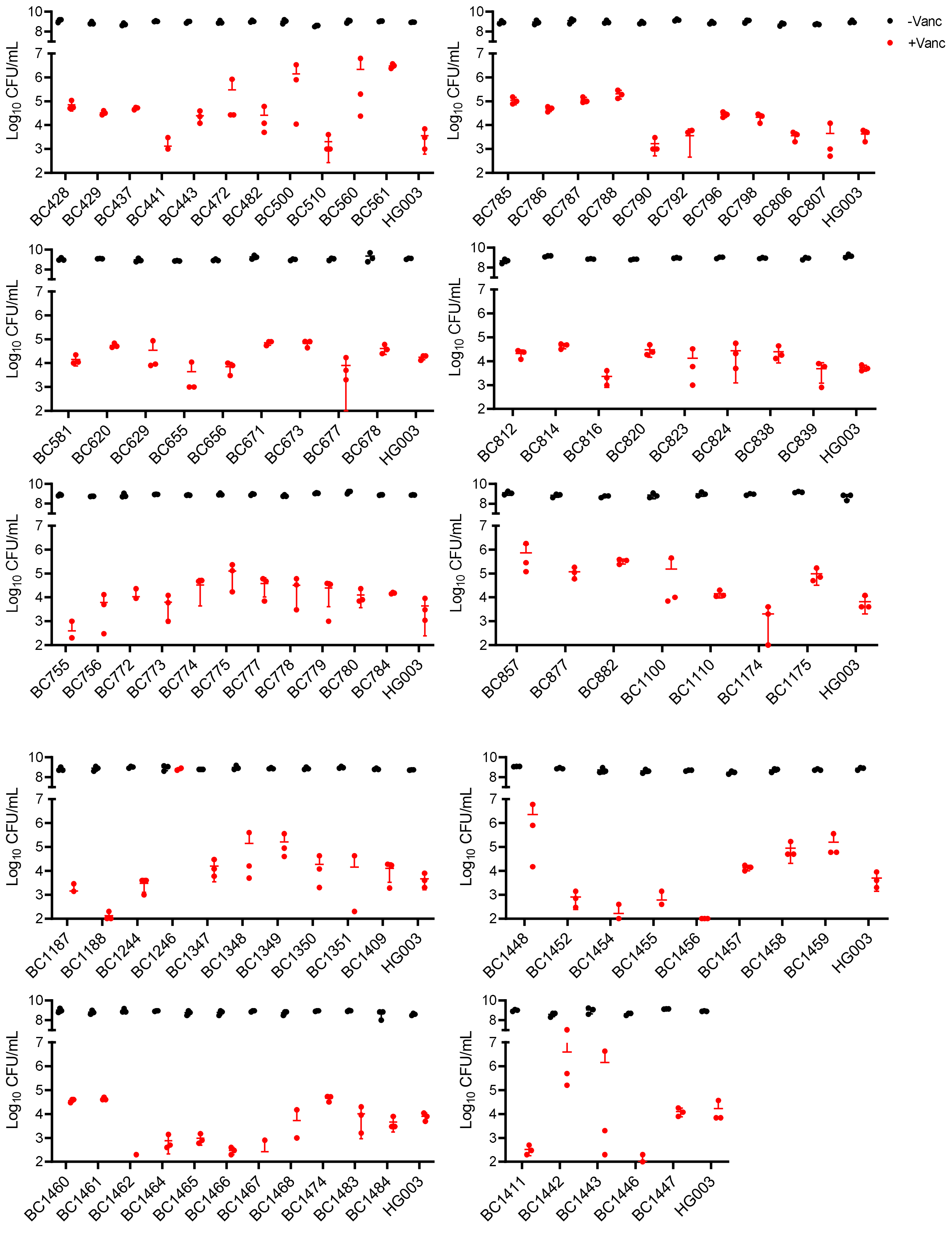

-Vanc
+Vanc
Log10 CFU/mL
BC428 BC429 BC437 BC441 BC443 BC472 BC482 BC500 BC510 BC560 BC561 HG003
BC785 BC786 BC787 BC788 BC790 BC792 BC796 BC798 BC806 BC807 HG003
BC581 BC620 BC629 BC655 BC656 BC671 BC673 BC677 BC678 HG003
BC812 BC814 BC816 BC820 BC823 BC824 BC838 BC839 HG003
BC755 BC756 BC772 BC773 BC774 BC775 BC777 BC778 BC779 BC780 BC784 HG003
BC857 BC877 BC882 BC1100 BC1110 BC1174 BC1175 HG003
BC1187 BC1188 BC1244 BC1246 BC1347 BC1348 BC1349 BC1350 BC1351 BC1409 HG003
BC1448 BC1452 BC1454 BC1455 BC1456 BC1457 BC1458 BC1459 HG003
BC1460 BC1461 BC1462 BC1464 BC1465 BC1466 BC1467 BC1468 BC1474 BC1483 BC1484 HG003
BC1411 BC1442 BC1443 BC1446 BC1447 HG003

Supplementary figure 4. The clinical MRSA isolates have different capacities to form antibiotic-tolerant cells. The clinical isolates were treated with 20 μg/mL vancomycin for 16 h (red circles) and the survival cells were enumerated. Black circles indicate growth without vancomycin. Strain HG003 was tested in parallel for comparison in each experiment. The bars represent mean ± SD (n = 3).

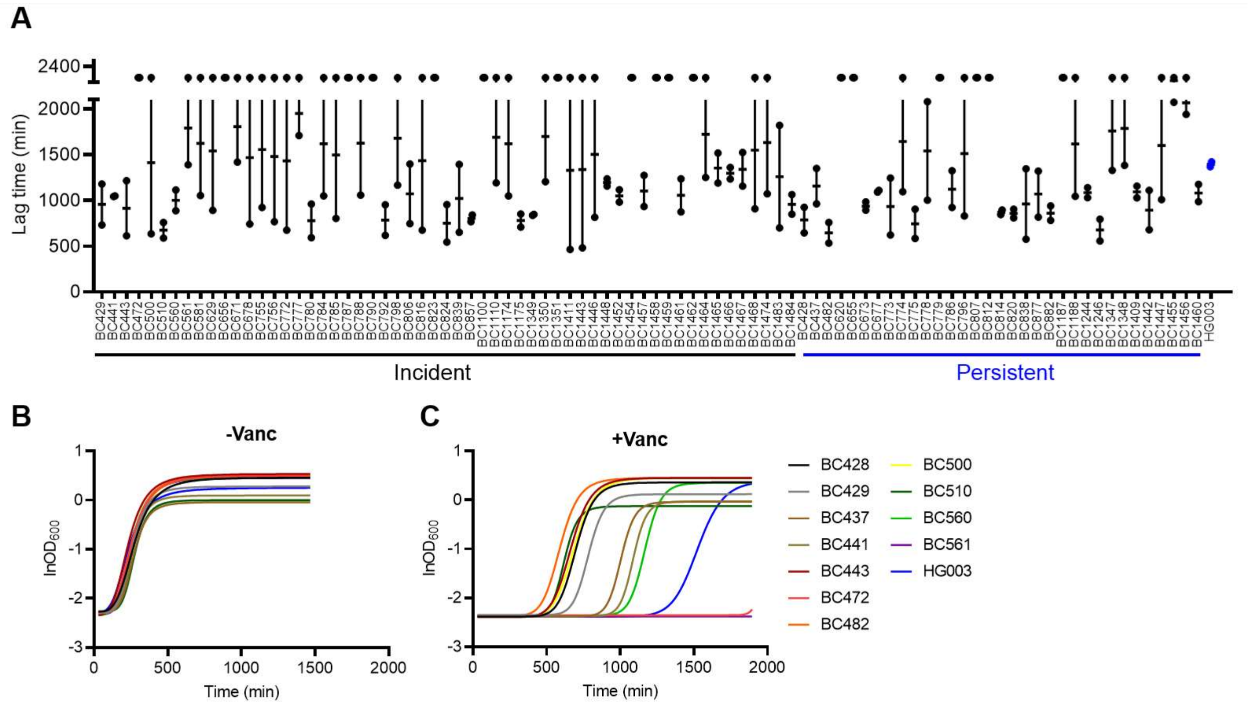

Supplementary figure 5. The recovery time (PAE) is highly variable in many MRSA isolates after vancomycin treatment. (**A**) Post-antibiotic lag times of the clinical isolates. Vancomycin-treated isolates were grown in an antibiotic-free medium to measure their growth for 36 h. The lag times were plotted as the maximum (2,190 min) if no growth was observed. The bars represent mean ± SD (n = 2). The lag times of untreated isolates were comparable to each other (**B**), while they became variable after vancomycin exposure (**C**). Wild-type HG003 was included as a reference strain (blue).
